# Supplementary material for: Role and mechanism of NCAPD3 in promoting malignant behaviors in gastric cancer
Source: Front Pharmacol. 2024 Apr 22;15:1341039. doi: 10.3389/fphar.2024.1341039 (PMC11070777; doi:10.3389/fphar.2024.1341039)
Supplement: Supplementary file 11 [file DataSheet2.ZIP › GSEA/Oncogenic signature/my_analysis.Gsea.1599461962066/CYCLIN_D1_KE_.V1_UP.html]

Details for gene set CYCLIN\_D1\_KE\_.V1\_UP[GSEA]

|  || Dataset | filtered\_dataset.sample\_info.cls#WT\_versus\_NCAPD3\_MUT |
| Phenotype | sample\_info.cls#WT\_versus\_NCAPD3\_MUT |
| Upregulated in class | NCAPD3\_MUT |
| GeneSet | CYCLIN\_D1\_KE\_.V1\_UP |
| Enrichment Score (ES) | -0.41637045 |
| Normalized Enrichment Score (NES) | -1.9012207 |
| Nominal p-value | 0.0078125 |
| FDR q-value | 0.031544693 |
| FWER p-Value | 0.253 |
Table: GSEA Results Summary

  

Fig 1: Enrichment plot: CYCLIN\_D1\_KE\_.V1\_UP      
 Profile of the Running ES Score & Positions of GeneSet Members on the Rank Ordered List

  

| SYMBOL | TITLE | RANK IN GENE LIST | RANK METRIC SCORE | RUNNING ES | CORE ENRICHMENT || 1 | 3304 | HSPA1B | 207 | 0.669 | -0.0878 | No |
| 2 | 1021 | CDK6 | 324 | 0.579 | -0.1184 | No |
| 3 | 3607 | FOXK2 | 522 | 0.461 | -0.2180 | No |
| 4 | 1119 | CHKA | 670 | 0.390 | -0.2881 | No |
| 5 | 2114 | ETS2 | 703 | 0.375 | -0.2770 | No |
| 6 | 1984 | EIF5A | 846 | 0.259 | -0.3555 | No |
| 7 | 6237 | RRAS | 869 | -0.280 | -0.3458 | No |
| 8 | 6273 | S100A2 | 942 | -0.365 | -0.3643 | No |
| 9 | 9314 | KLF4 | 972 | -0.389 | -0.3497 | No |
| 10 | 833 | CARS | 1022 | -0.420 | -0.3466 | No |
| 11 | 7204 | TRIO | 1120 | -0.494 | -0.3713 | Yes |
| 12 | 1263 | PLK3 | 1134 | -0.502 | -0.3349 | Yes |
| 13 | 6310 | ATXN1 | 1177 | -0.547 | -0.3151 | Yes |
| 14 | 440 | ASNS | 1232 | -0.609 | -0.2984 | Yes |
| 15 | 2355 | FOSL2 | 1262 | -0.648 | -0.2602 | Yes |
| 16 | 861 | RUNX1 | 1293 | -0.687 | -0.2191 | Yes |
| 17 | 3084 | NRG1 | 1320 | -0.739 | -0.1704 | Yes |
| 18 | 7706 | TRIM25 | 1357 | -0.817 | -0.1219 | Yes |
| 19 | 5794 | PTPRH | 1368 | -0.854 | -0.0512 | Yes |
| 20 | 5630 | PRPH | 1371 | -0.885 | 0.0280 | Yes |
Table: GSEA details [plain text format]

  

Fig 2: CYCLIN\_D1\_KE\_.V1\_UP      
 Blue-Pink O' Gram in the Space of the Analyzed GeneSet

  

Fig 3: CYCLIN\_D1\_KE\_.V1\_UP: Random ES distribution      
 Gene set null distribution of ES for **CYCLIN\_D1\_KE\_.V1\_UP**
